# The effects of static and dynamic stretching on deep fascia stiffness: a randomized, controlled cross-over study

**DOI:** 10.1007/s00421-024-05495-2

**Published:** 2024-04-30

**Authors:** Konstantin Warneke, Thomas Rabitsch, Patrik Dobert, Jan Wilke

**Affiliations:** 1 Institute of Human Movement Science, Sport and Health, University of Graz, 8020 Graz, Austria; 2 Institute of Sport Science, Alpen-Adria University Klagenfurt, 9020 Klagenfurt am Wörthersee, Austria; 3 Department of Neuromotorics and Movement, University of Bayreuth, 95447 Bayreuth, Germany

**Keywords:** Connective tissue, Flexibility, Range of motion, Fascial properties

## Abstract

**Aim:**

Previous stretching studies mostly investigated effects on the skeletal muscle but comprehensive explorations regarding the role of the connective tissue are scarce. Since the deep fascia has been demonstrated to be sensitive to mechanical tension, it was hypothesized that the fascia would also respond to stretching, contributing to enhanced range of motion (ROM).

**Methods:**

Forty (40) recreationally active participants (male: *n* = 25, female: *n* = 15) were included in the randomized controlled cross-over trial and allocated to different groups performing 5 min static (STAT) or dynamic (DYN) plantar flexor stretching or control condition (CC) in a random order. Pre- and immediately post-intervention, muscle and fascia stiffness, as well as muscle and fascia thickness were measured using high-resolution ultrasound and strain elastography. ROM was assessed in the ankle joint via the knee to wall test (KtW) and goniometer.

**Results:**

STAT reduced both, muscle and fascia stiffness (*d* = 0.78 and 0.42, *p* < 0.001, respectively), while DYN did not reduce stiffness compared to the control condition (*p* = 0.11–0.41). While both conditions showed significant increases in the KtW (*d* = 0.43–0.46, *p* = 0.02–0.04), no significant differences to the CC were observed for the isolated ROM testing (*p* = 0.09 and 0.77). There was a small correlation between fascia stiffness decreases and ROM increases (*r* = − 0.25, *p* = 0.006) but no association was found between muscle stiffness decreases and ROM increases (*p* = 0.13–0.40).

**Conclusion:**

Our study is the first to reveal stretch-induced changes in fascia stiffness. Changes of fascia`s but not muscle`s mechanical properties may contribute to increased ROM following stretching.

## Introduction

A vast amount of research has explored the effects of stretching on range of motion (ROM) (Behm et al. 2023; Konrad et al. 2023). While static stretching is superior in enhancing ROM in the long-term (Konrad et al. 2023), dynamic and static stretching are similarly effective with regard to acute application (Cai et al. 2023). Available explanatory approaches for the flexibility increases following stretching are twofold. Many researchers attributed the acute and chronic ROM enhancements to neurophysiological changes modifying the pain threshold (Freitas et al. 2018; Konrad et al. 2022; Konrad and Tilp 2014). Indeed, Stove et al. (2021) demonstrated that stretch tolerance is linked to endogenous pain inhibition which, in turn, may be triggered by stretch application. However, recent data suggests additional morphological or structural stretching effects, i.e. in muscle- and/or muscle–tendon adaptations (Opplert and Babault 2018; Takeuchi et al. 2023a, b). For instance, it is assumed that a decrease in the mechanical stiffness would allow for larger muscle–tendon elongation and a consecutively increased ROM. This idea is supported by a systematic review of Takeuchi et al. (2023a) which demonstrated chronic static stretching to reduce muscle stiffness and acute muscle–tendon unit stiffness decreases (Takeuchi et al. 2023a, b).

While a plethora of studies focused on stretching and muscle–tendon properties (Cai et al. 2023; Takeuchi et al. 2023a, b; Takeuchi et al. 2023a), there is a paucity of research addressing its effect on non-tendinous connective tissues such as the deep fascia. This is noteworthy because it has been shown that fascia can modify its stiffness by means of contractile cell activity regulation, thixotropic tissue behavior and fluctuations of the water content (Schleip et al. 2019; Wilke et al. 2019a, b). For instance, in an in vitro-experiment, Schleip et al. (2012) applied 15 min static stretches to mice lumbodorsal fasciae and observed an increase in tissue stiffness. In addition to being capable of changing its mechanical stiffness, fascia has a high nociceptive capacity. Barry et al. (2015) reported the deep fascia of the lower limb and the lower back region to have a threefold higher nerve density than the underlying muscles and most of the nerve fibers contained substance P and/or CGRP (calcitonin gene-related peptide). Schilder et al. (2014) applied nociceptive stimuli to human skeletal muscle and deep fascia. Injections with hypertonic saline solution into the connective tissue induced substantially stronger pain responses than those into the muscle. Taken together, due to its capacities to modify tissue stiffness and to produce pain sensations, fascia may play a role in stretching effects.

To the best of our knowledge, no study has yet explored the effects of stretching on the mechanical properties of fascia. Specifically, it is unknown if (a) stretching can modify fascial stiffness under in vivo conditions in humans, (b) static and dynamic stretching have different impacts on fascia and (c) potential stiffness changes are related to ROM improvements. Our study aimed to address these questions. We hypothesized that stretching, in addition to increasing flexibility, would reduce fascia stiffness as well as that static stretching would have larger effects due to the higher net stimulus duration. In addition, we assumed that potential stiffness reductions would correlate with flexibility improvements.

## Methods

### Ethics and experimental design

A randomized crossover study with three conditions (5 min static calf stretching (STAT), 5 min dynamic calf stretching (DYN), 5 min inactive control (CON)) was performed. Before and immediately after (< 60 s after releasing the stretch in STAT and DYN) each session, we investigated soft tissue stiffness using ultrasound elastography and ankle joint ROM using the knee-to-wall test and a goniometer. Between conditions, a washout-phase of 2 days was used to prevent carryover effects. The study was conducted under consideration of the Declaration of Helsinki and approved by the local ethical review board (No. 2023-037). All participants provided written informed consent.

### Participants

We performed an a priori sample size calculation for a repeated-measures ANOVA (3 × 2) using G-Power (Version 3.1., Heinrich-Heine-Universität Düsseldorf, Düsseldorf, Germany). Since no previous study investigated the effects of stretching on fascia stiffness, we computed Cohen`s d of muscular stiffness pre-post changes from Konrad et al. (2017). Assuming *d* = 0.5 (*f* = 0.25), a power of 80% and a significance level of 0.05, a minimal sample size of *n* = 36 was deemed sufficient. To account for potential dropouts, 40 healthy, trained participants (male: *n* = 25, 27.7 ± 5.5 years, height: 179.4 ± 5.3 cm, mass: 79.2 kg, 10.3 ± 2.2 h of athletic activity per week, females: *n* = 15, age: 24.6 ± 3.4 years, height: 196.0 ± 3.3 cm, weight: 67.3 ± 5.5 kg, 6.9 ± 4.4 h of athletic activity per week) were recruited at the local sports university campus. Participants with any kind of lower limb injury within the last six months, unhealed musculoskeletal complaints, or a reported risk of artery disorders were excluded from the study. Inclusion criteria comprised an age between 18 and 40 years and a minimum weekly sporting activity of 3 h.

### Intervention

**Fig. 1:**
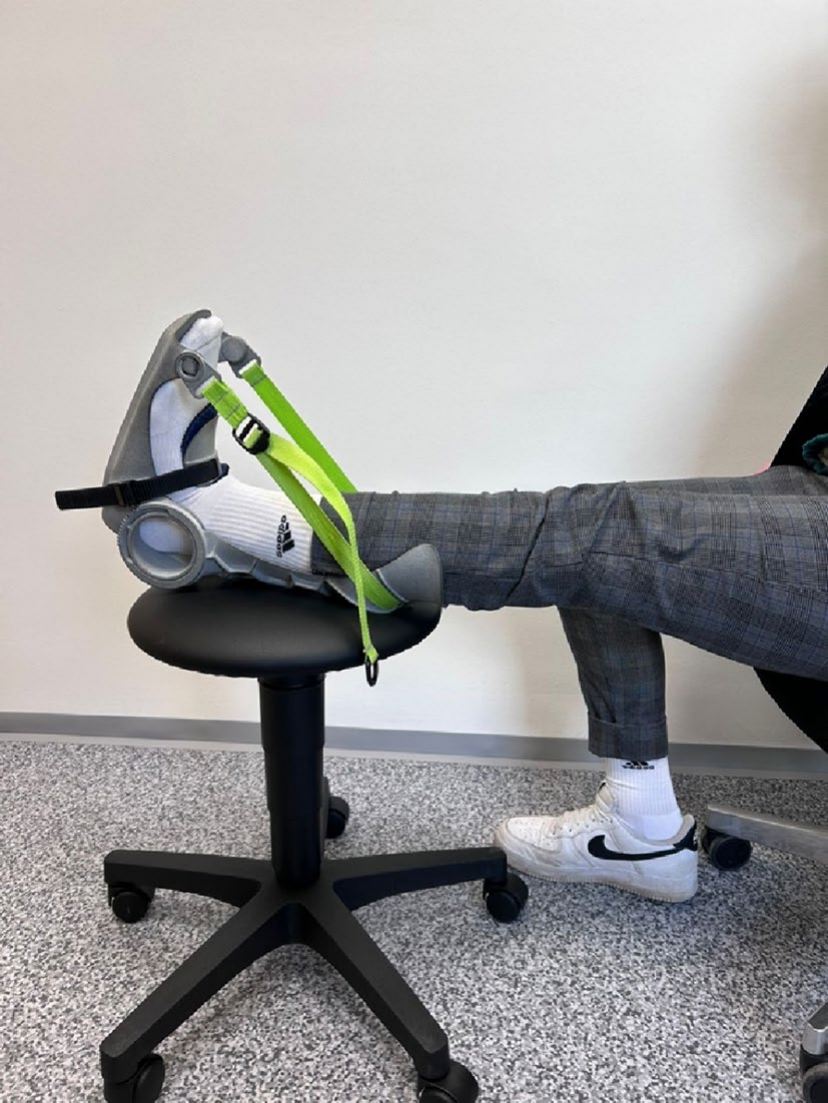
The stretching intervention using the calf muscle orthosis built for standardized stretching, the reached ROM was measured by the included goniometer

In the STAT and DYN conditions, calf stretching was performed for five minutes. This duration was chosen because previous research demonstrated decreases in muscle–tendon stiffness with an increased passive torque in the end ROM only after long as opposed to short stretch duration (Nakamura et al. 2013). We used a stretching device evaluated by Warneke et al. (2022a, b) to ensure a standardized, constant and easy-to-apply stretch. For STAT, the participants’ ankle was fixed in the maximal dorsiflexed position to ensure constant-angle stretching. In detail, while the participant sat on a chair (hip flexion angle 90°) with extended knee joint, an investigator pushed the foot fixed in the device into maximal dorsiflexion and this joint angle was locked (Fig. 1). The maximum angle was determined based on the participants feedback of maximal tolerable stretching pain. The angle selected in this assessment was used as the starting angle of the stretching intervention. During the 5 min stretch, the participant was instructed to place the leg with the stretching device on a chair. The leg and the back were to be held in a straight position to maximize the stretch on the plantar flexors (Fig. 1).

As indicated, the stretch device was also used for DYN. However, instead of locking the ankle angle in maximal dorsiflexion, dynamic stretching was applied performing cyclic soft tissue elongations without a static phase until maximal discomfort was reported by the participants. Dynamic stretching was performed passively, as one investigator alternatingly pushed the foot into maximal dorsiflexion and released the pressure with a cadence of 1–0–1–0 (seconds) for 5 min. This resulted in a total of 150 stretch cycles. Each cycle consisted of pushing the foot into maximal dorsiflexion within one second. After reaching maximal discomfort and without a static phase, the pressure was released for one second to get back in (at least) the neutral 0 at 90° ankle joint position. Afterwards, the investigator applying the stretch again pushed the foot into maximal dorsiflexion within 1 s. The angle reached at maximal stretching discomfort in the initial repetitions was document by an investigator not performing the stretch in order to ensure reaching the same dorsiflexion angle in each repetition. The CON condition consisted of 5 min inactive sitting on a chair without any intervention or instruction.

### Outcomes

#### Fascia and muscle stiffness and thickness

Before testing started, the participants performed 2 × 30 s jumping jacks with a rest of 30 s in between as a light warm-up routine. To examine morphological and mechanical tissue properties, a high-resolution ultrasound device (Ecube15, Alpinion, Germany) was used. A linear 5–24 MHz transducer (width: 15 cm) was placed on the muscle belly of the gastrocnemius medialis, which was palpated at about 25–30% of the distance between the most lateral point of the articular cleft of the knee to the lateral top of the malleolus lateralis. To ensure identical measurement locations on the different appointments, the position of the transducer was marked on the skin of the participants with a water-resistant pen. The participant was instructed to lie down in a prone position on a treatment table. The legs were relaxed with the feet hanging over the end of the table to avoid any contraction of the calf muscles. The knee joint rested in an extended position, without performing any contractions, as previously described in Warneke et al. (2022a, b).

**Fig. 2:**
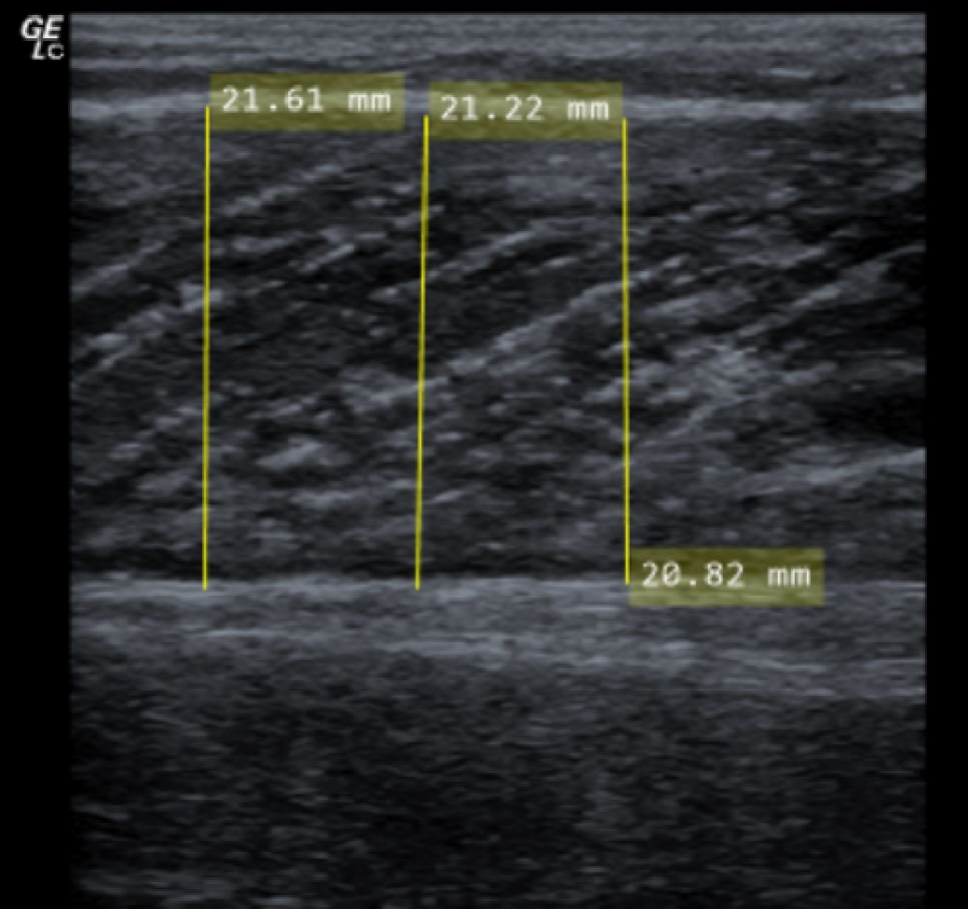
The muscle thickness assessment in the plantar flexors

Three static ultrasound images of the calf were obtained. Deep fascia thickness was measured as indicated in Fig. 2. Similarly, to track potential increases due to swelling, muscle thickness was measured as marked in Fig. 2. Thickness measurements were performed at three (proximal, central and distal) equidistant locations per image. The mean of the six measurements (three measurements in each of the three images) was used for analysis. This procedure has been shown to be reliable and valid (ICC = 0.95–0.97) (Warneke et al. 2022a, b).

Muscle- and fascia stiffness were determined using strain elastography. Briefly, the technique applies a light compression stimulus to the measured tissue and tracks its speed of propagation. A stiffness ratio is calculated between the stiffness of the target region (deep fascia, skeletal muscle) and a reference region. This reference was a gel pad with a constant stiffness, positioned on the skin of the participant. To guarantee consistency of gel pad stiffness, room temperature was held constant at 20 °C. Shimoyama et al (2021), Chino et al. (2014) and Brage et al. (2019) stated strain elastography for muscle- and tendon assessments with and without gel pad to be reliable with ICCs > 0.7–0.89. The mean of two consecutive measurements with three regions of interests for muscle stiffness and three regions of interest for fascia stiffness (thus six measurements for muscle- and six measurements for fascia stiffness) was calculated and used for further processing. Ultrasound measurements were performed by an investigator with an experience of more than 4000 ultrasound muscle assessments.

### Range of motion

Range of motion of the ankle was measured both with an isolated test and a functional test. The isolated ROM assessment was performed using a goniometer attached to a stretching device, as this procedure was previously described to be highly reliable (ICC = 0.995, (Warneke et al. 2022a, b). Participants positioned their foot on a chair, while fixed in the stretching device. Keeping the knee of the participant extended, the investigator pushed the foot into the maximally dorsiflexed position, until the point of maximal tolerable discomfort was reported. The ankle angle [°] was then read from the attached goniometer. This procedure was performed twice and the mean of both measurements was used for further calculations.

The functional knee joint ROM measurement was performed using the knee-to-wall-test which has been frequently used and validated in previous trials (Silva et al. 2018; Wyndow et al. 2016). Standing in the split stance on a measurement tape, the participants were instructed to push their knee towards the wall as far as possible, aiming to keep the heel on the ground. As early as the heel lifted off, the measurement was finished and the distance of the patella to the wall (cm) was documented. The mean of two trials was used for analysis. The knee-to-wall test was stated as a reliable procedure with an ICC of 0.99 (Warneke et al. 2022a, b).

### Statistical analysis

Data analysis was performed using Jamovi (the JAMOVI project). Normal distribution and homogeneity of variance of the collected data were explored using the Shapiro–Wilk test and the Levene’s test, respectively. Reliability was calculated via the two-way agreement intraclass correlation coefficient (ICC). To identify baseline differences between the conditions, a one-way analysis of variance (ANOVA) was performed for each parameter.

To examine differences in tissue stiffness and thickness between conditions, two-way ANOVAs with repeated measures (3 conditions: STAT/DYN/CON × 2 time points: baseline/post) and the Scheffé test as post-hoc test were calculated. Effect sizes of the ANOVA were interpreted as small (*η*^2^ < 0.06), moderate (*η*^2^ = 0.06– < 0.14), or large (*η*^2^ ≥ 0.14), while inter-group pre-post differences of the post-hoc test were interpreted as trivial (*d* < 0.2), small (*d* < 0.5), moderate (*d* = 0.5– < 0.8), or large (*d* ≥ 0.8), based on the classification of Cohen (1988). To explore relationships between stiffness and range of motion changes, univariate Pearson correlation coefficients were calculated and interpreted as described for Cohen’s d. To investigate differences of fascia and muscle stiffness changes, a *t* test for paired samples was performed. The level of significance was set to *α* < 0.05.

## Results

During the study period, one participant dropped-out due to an anterior cruciate ligament rupture, but this was not related to the study. All other participants completed the assignments as scheduled. The reliability of the muscle and fascia measurements ranged from ICC = 0.7–0.97 for ultrasound measurements and ICC = 0.98–1 for flexibility testing (Table 1).

**Table 1:** Intraclass correlation coefficients for the measured parameters

Parameter	ICC (95% CI)
Muscle stiffness (ratio)	0.844 (0.78–0.90)
Fascia stiffness (ratio)	0.702 (0.60–0.78)
Muscle thickness	0.955 (0.94–0.97)
Fascia thickness	0.940 (0.91–0.96)
ROM (knee to wall)	1.0 (1.0–1.0)
ROM (goniometer)	0.98 (0.97–0.99)

None of the measured parameters showed baseline differences (*p* > 0.05). The descriptive statistics as well as the results of the two-way ANOVA with repeated measures are provided in Table 2.

**Table 2:** Tissue stiffness, thickness, and range of motion before and after STAT, DYN and CON

Parameter	Group	Pre (M ± SD)	Post (M ± SD)	Main effect (time)	Interaction (time × condition)
Muscle stiffness	STAT DYN CON	0.30 ± 0.10 0.30 ± 0.11 0.29 ± 0.09	0.22 ± 0.08 0.27 ± 0.12 0.30 ± 0.09	F (114,1) = 23.2 *p* < 0.001 $\eta_{{\text{p}}}^{2}$ = 0.17	F (114,2) = 11.6 *p* < 0.001 $\eta_{{\text{p}}}^{2}$ = 0.17
Muscle thickness	STAT DYN CON	6.37 ± 1.08 6.44 ± 1.10 6.35 ± 1.16	6.34 ± 1.09 6.28 ± 1.20 6.32 ± 1.16	F (114,1) = 4.14 *p* = 0.04 $\eta_{{\text{p}}}^{2}$ = 0.04	F (114,2) = 1.46 *p* = 0.24 $\eta_{{\text{p}}}^{2}$ = 0.03
Fascia stiffness	STAT DYN CON	0.26 ± 0.08 0.24 ± 0.07 0.25 ± 0.07	0.18 ± 0.06 0.21 ± 0.10 0.24 ± 0.07	F (114,1) = 23.85 *p* < 0.001 $\eta_{{\text{p}}}^{2}$ = 0.17	F (114,2) = 8.00 *p* < 0.001 $\eta_{{\text{p}}}^{2}$ = 0.12
Fascia thickness	STAT DYN CON	0.33 ± 0.11 0.33 ± 0.11 0.33 ± 0.10	0.33 ± 0.11 0.33 ± 0.10 0.32 ± 0.09	F (114,1) = 0.492 *p* = 0.484 $\eta_{{\text{p}}}^{2}$ = 0.004	F (114,2) = 1.269 *p* = 0.285 $\eta_{{\text{p}}}^{2}$ = 0.02
Knee to wall	STAT DYN CON	12.2 ± 3.2 12.0 ± 3.3 12.3 ± 3.1	12.6 ± 3.13 12.5 ± 3.4 12.0 ± 3.2	F (114,1) = 3.84 *p* = 0.052 $\eta_{{\text{p}}}^{2}$ = 0.03	F (114,2) = 5.00 *p* = 0.008 $\eta_{{\text{p}}}^{2}$ = 0.08
Goniometer	STAT DYN CON	8.74 ± 1.82 8.65 ± 1.60 8.97 ± 1.88	9.73 ± 2.2 9.08 ± 1.7 9.2 ± 1.89	F (114,1) = 30.67 *p* < 0.001 $\eta_{{\text{p}}}^{2}$ = 0.21	F (114,2) = 4.78 *p* = 0.010 $\eta_{{\text{p}}}^{2}$ = 0.08

### Tissue thickness

There was a small main effect for time (*p* = 0.04, $\eta_{{\text{p}}}^{2}$ = 0.04), indicating a decrease in muscle thickness. No condition × time interaction was found. Fascia thickness was unchanged (*p* > 0.05).

### Tissue stiffness

**Fig. 3:**
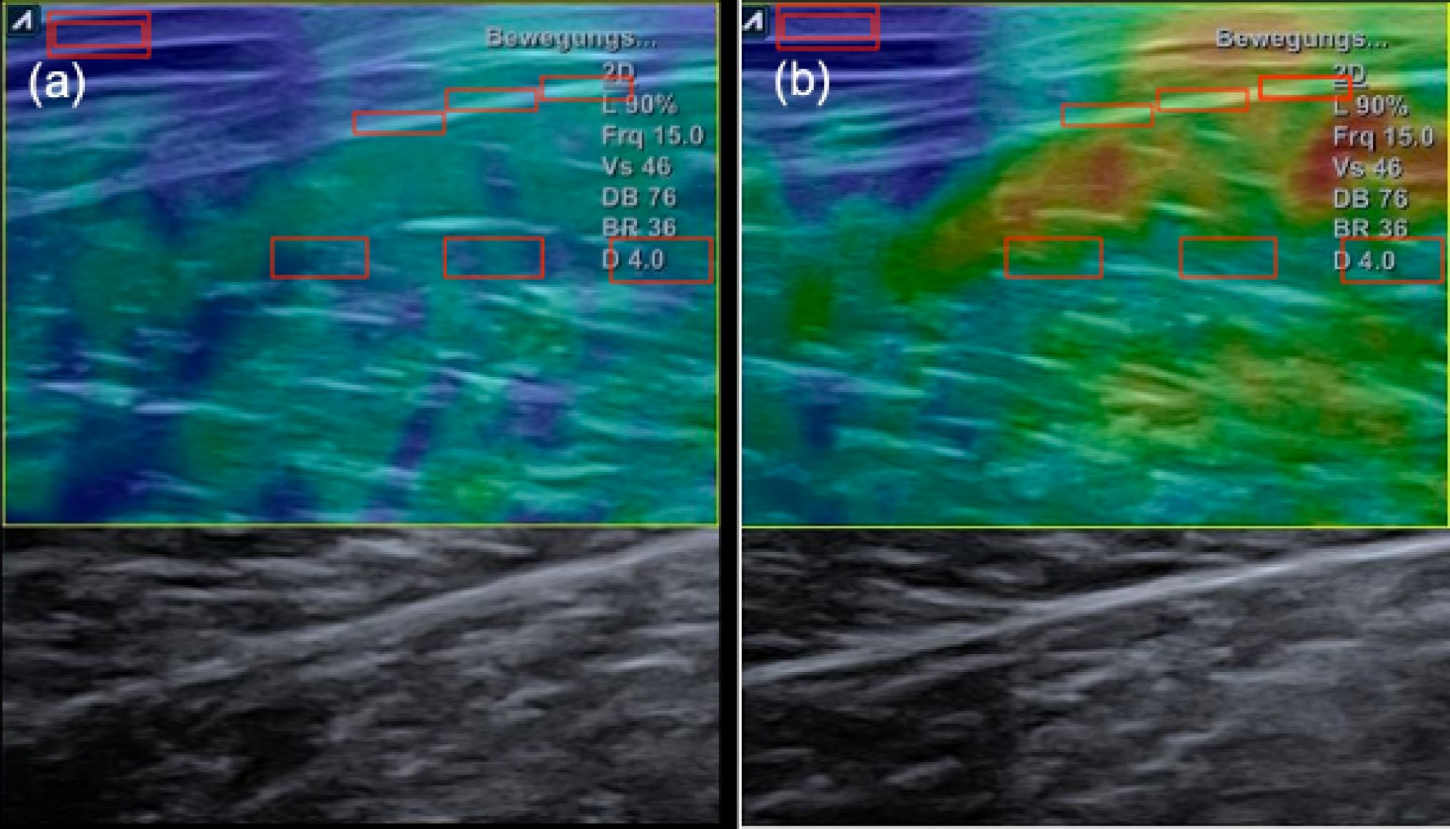
Changes of muscle- and fascia stiffness ratios from pre (left) to post (right). While blue color represents high stiffness, green, yellow, and red, in this order, represent lower stiffness. The gel pad with constant stiffness, which was used as a reference, can be seen in the upper left corner in both images

There was a large decrease (main effect for time: *p* < 0.001, $\eta_{{\text{p}}}^{2}$ = 0.13) in fascia stiffness and a large time × group interaction (*p* < 0.001, $\eta_{{\text{p}}}^{2}$ = 0.17). The Scheffé test showed differences between STAT and DYN (*p* = 0.04, d = 0.64), and between STAT and CON (*p* < 0.001, *d* = 0.42), however, no significant difference between DYN and CON was found (*p* = 0.41) (Fig. 3b). Therefore, static stretching induced fascia stiffness changes, while effects induced via dynamic stretching did not change stiffness.

**Fig. 4:**
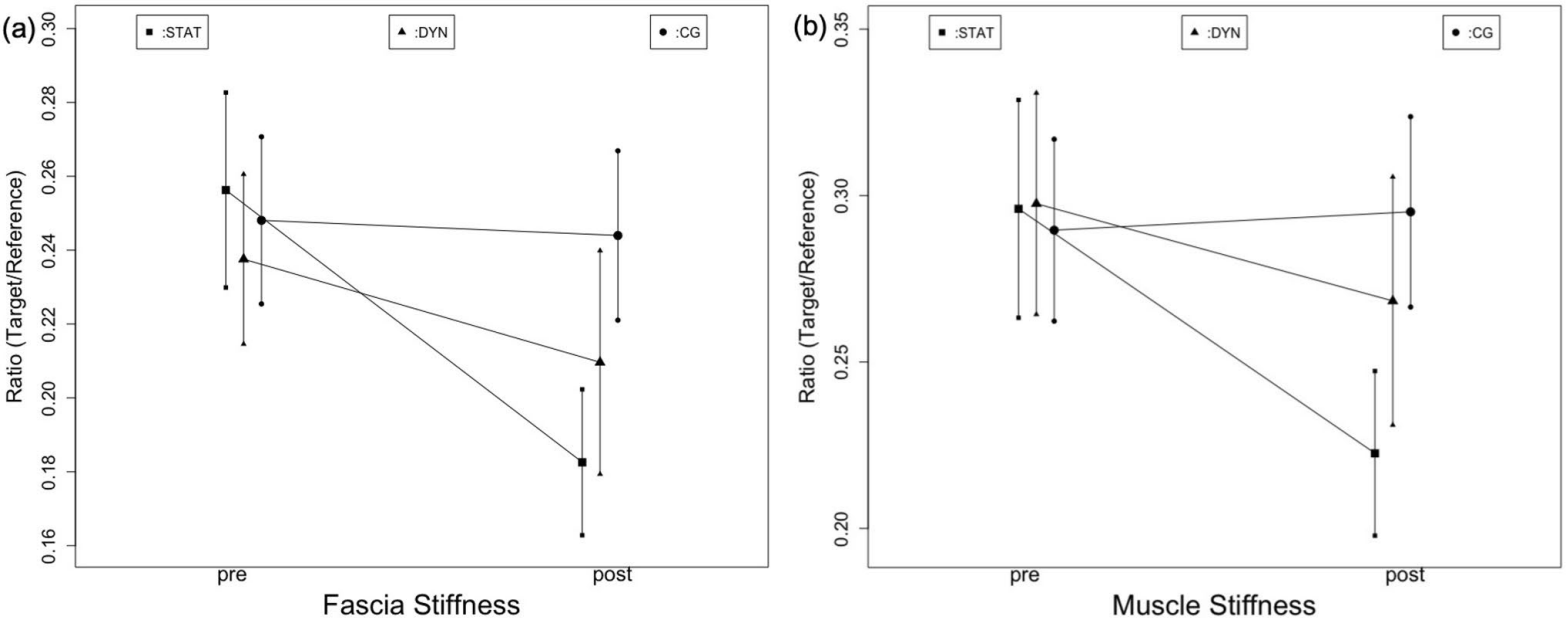
Pre–post-changes of fascia stiffness (**a**) and muscle stiffness (**b**) following static (STAT), dynamic (DYN) and the control condition (CG)

We also found a large decrease (main effect for time, *p* < 0.001, $\eta_{{\text{p}}}^{2}$ = 0.17) in muscle stiffness and a large time × group interaction (*p* < 0.001, $\eta_{{\text{p}}}^{2}$ = 0.17). Post hoc testing revealed significant differences between STAT and DYN (*p* = 0.03, *d* = 0.43) and STAT and CON (*p* < 0.001, *d* = 0.78). No difference was observed between DYN and CON (*p* = 0.11) (Fig. 3a). Similar to the deep fascia, results show static stretching to decrease stiffness, while dynamic stretching showed no significant changes (Fig. 4).

### Isolated ROM

Ankle ROM measured using goniometer showed large increases (main effect for time: *p* < 0.001, $\eta_{{\text{p}}}^{2}$ = 0.21), with a moderate group × time interaction (*p* = 0.008, $\eta_{{\text{p}}}^{2}$ = 0.08). Post-hoc testing was only significant for STAT vs. CON (*p* = 0.014, *d* = 0.48). No difference was found between STAT and DYN (*p* = 0.09) as well as DYN and CON (*p* = 0.77). Therefore, using goniometer testing, static stretching, but not dynamic stretching increased flexibility.

### Functional ROM

The ROM increase measured via the knee to wall test marginally failed to reach the level of significance (*p* = 0.05, $\eta_{{\text{p}}}^{2}$ = 0.03), but a time × group interaction was found (*p* = 0.008, $\eta_{{\text{p}}}^{2}$ = 0.08). The Scheffé test revealed a difference for both, STAT and DYN compared to CON (*p* = 0.02, *d* = 0.46, *p* = 0.04, *d* = 0.43, respectively) indicating a ROM increase via both stretching techniques. No difference was found between STAT and DYN (*p* = 0.98).

### Association of stiffness and ROM changes

The stiffness ratios of muscle and deep fascia were not different for all conditions (*p* = 0.68). However, a small association of fascia stiffness changes and ROM improvements, measured via the goniometer, was detected (*r* = − 0.25, *p* = 0.006). Muscle stiffness changes did not correlate with ROM improvements (*p* > 0.05).

## Discussion

To the best of our knowledge, this study was the first to explore deep fascia’s mechanical properties following stretching under in vivo conditions. Both STAT and DYN induced decreases of stiffness and the magnitudes were similar to the reductions in the skeletal muscle. Regarding the latter, our results accord with a previous trial of Konrad et al (2019) who found static and dynamic stretching to acutely reduce muscle stiffness. Yet, interestingly, with *d* = 0.42 (fascia) and *d* = 0.78 (muscle), we found larger effects of static stretching on stiffness and ankle ROM; hence, static stretch may be preferred by athletes and practitioners if aiming to target one of these outcomes.

Fascia stiffness and range of motion.

While it is widely known that stretching acutely increases ROM (Behm et al. 2023), the underlying mechanisms are not finally clarified. Although neuronal aspects such as a reduced pain perception (Cabido et al. 2014; Konrad et al. 2022) as well as mechanical aspects including muscle-, tendon- or muscle–tendon unit stiffness changes (Opplert and Babault 2018; Takeuchi et al. 2023a, b) have been discussed, the explanatory approaches provided hitherto neglected the potential role of the deep fascia. While our results are in line with previous studies indicating stretch-induced changes in muscle stiffness (Konrad et al. 2017; Zhou et al. 2019), biological systems require a holistic point of view, the skeletal muscle should not investigate as an isolated element of the locomotor system (Turrina et al. 2013). Nevertheless, to date, even though extensively described to be receptive to mechanical stimuli (Schleip et al. 2005) research exploring exercise effects on fascial structure is still scarce. Interestingly, the only available studies, investigating the effects of exercise (maximal eccentric exercise) on the deep fascia found stiffness changes and thickness increases in the fascia, while the muscle remained unaffected (Wilke et al. 2022). Of note, such exclusive reaction of the fascia was not found in this study as we did not find any difference between muscle- and fascia stiffness reductions. Although further studies, using different stretch durations, additional follow-up measurements, and different outcomes (i.e. fascial layer sliding) are needed, it may be carefully speculated that eccentric loading but not stretching can selectively stimulate the deep fascia.

While both neural and structural effects jointly contribute to the ROM increases following stretching, it is mostly agreed on that neural mechanisms such as an improved stretch tolerance represent the primary factor. Notwithstanding, peripheral nerve stiffness has been shown to strongly correlate with ROM (*r* = 0.57; (Andrade et al. 2018)). In accordance with previous research controversy discussing the relationship between ROM and muscle stiffness (Miyamoto et al. 2018; Nakamura et al. 2021a, b, c), our study showed that muscle stiffness changes does not seem associated with ROM increases. In contrast, we found a correlation of fascia stiffness reductions and ROM improvements. Although the magnitude of this relation was small (*r* = − 0.25), this finding fits with research demonstrating a high sensory capacity of fascia (Barry et al. 2015; Schilder et al. 2014). In view of the exploratory nature of our study and because causality cannot be inferred from our data, additional research further delineating the relevance of fascia in ROM improvement is needed.

In the literature, dynamic movements and bouncing-like action including a pre-stretch phase have been claimed to trigger fascial reconstruction (Schleip and Müller 2013). However, as collagen remodeling rather occurs in the long-term, this would rather be expected for chronic application. Interestingly, at least with reference to immediate effects, static stretching showed a higher impact on stiffness. When selecting exercise aiming to target the fascia, our study therefore reinforces the need to distinguish between acute and long-term effects and with regard to the former, static lengthening seems more appropriate to modify fascial stiffness.

In general, intensity and duration are suggested to moderate stretching -induced flexibility and stiffness alterations (Freitas et al. 2015; Nakamura et al. 2021a). Mechanical tension, measured via time under tension can be assumed to be higher using static compared to dynamic stretching. Nevertheless, while duration can be determined objectively, there are some concerns regarding stretching intensity. It is commonly quantified via the individual pain threshold (Nakamura et al. 2021b; Takeuchi et al. 2021; Warneke et al. 2022a) but Lim and Park (2017) questioned the objectivity and validity of this procedure. Accordingly, to counteract variations in the stretching stimulus, we a) used a goniometer to ensure a constant ankle angle during static stretching and b) controlled the reached ankle angle during dynamic stretching.

Participants reported higher pain when using static (7–8 on the numerical rating scale) compared to dynamic stretching (3–4). These differences could be attributed to the possibility of reaching a higher ROM during STAT due to relaxation effects. However, it is also possible that the strong pain sensation in STAT originated from the continuous stretch, while the DYN condition naturally included brief phases of recovery (lower net duration in the maximal ankle angle). In sum, it cannot be finally clarified whether STAT induced higher stretch intensities because no objective quantification of stretching force/resistive force of the muscle was carried out. Future studies may hence consider measuring passive resistance testing using a dynamometer.

Previous literature on muscle stiffness.

The hypothesis that the type of stretching, and with this, the duration or intensity of the induced stimulus could play a role for stiffness adaptations, has been discussed in previous literature. Zeleznik et al. (2024) reported only proprioceptive neuromuscular facilitation (PNF) stretching to reduce biceps femoris muscle stiffness, while static stretch increased flexibility only. In contrast, an earlier study by Hirata et al. (2020) showed stiffness reductions in response to static stretching, while Konrad et al. (2017) found PNF, static and ballistic stretching to decrease muscle stiffness of the plantar flexors. As a consequence, the muscle specificity with regard to the potential muscle specificity may be an additional variable to consider in future research. Interestingly, discrepancies could not only arise from different muscle groups. Zhou et al. (2019) found non-uniform stiffness changes in the gastrocnemius. While in the pre-test, the proximal region showed the highest and the mid-third of the muscle the smallest stiffness values, tests post-stretching revealed no significant stiffness differences within the three gastrocnemius muscle regions. In addition to the muscle or fascia location, age represents a potential effect modifier. Even though Hirata et al. (2020) showed superior effects of stretching in younger participants, gastrocnemius muscle stiffness reductions were also seen in older adults (Nakamura et al. 2024), which opens research gaps for further studies investigating the role of fascia property responses including stiffness and thickness and its role in acute ROM increases via different types of stretching in different cohorts (Wilke et al. 2019a). Regarding muscle stiffness, our findings are in accordance with Zeleznik et al. (2024) who found no significant correlations between muscle stiffness decreases and ROM increases. However, since we found ROM increases to be slightly correlated with fascia stiffness, solely relating acute ROM improvements to neuronal reactions such as enhanced stretch tolerance needs further investigation.

Muscle- and fascia thickness.

Although we observed a statistically significant reduction in muscle thickness, with *d* = 0.03–0.14, effect sizes were trivial. Accordingly, assessed muscle thickness changes in the reported range (0.03–0.16 cm) can be speculated to be attributable to daily variations or measurement errors due to reliability and objectivity concerns of using ultrasound diagnostics.

## Limitations

There are some limitations to be considered when interpreting our results. First, although frequently used, ultrasound can have some methodological shortcomings, including issues of objectivity due to subjective influence of applied probe pressure to the skin, the used angle as well as location of measurement. To counteract this, extensive piloting measurements were made until achieving consistent results. Furthermore, all measurements were performed by the same experienced investigator to ensure highest consistency as possible. Regarding fascia stiffness ratios, the reliability with ICC ≥ 0.7 with a lower border of ICC = 0.6 is in line with current literature (Shimoyama et al. 2021), but could be considered comparatively low. Consequently, advanced strain elastography measurement protocols should be invested to improve reliability of the testing procedure in future studies. Especially since the only significant correlation was obtained between fascia stiffness and ROM improvements, future studies are required to confirm our findings. Second, although we did not find differences between muscle and fascia immediately post-intervention, this could well be the case at later time points as production of relevant fluids such as hyaluronic acid in the fascia and blood flow in the muscle and fascia might occur after removing the stretching stimulus (Hotta et al. 2018). Future studies may thus consider including additional follow-up measurements. Third, as indicated above, we did not quantify stretching pain (or other indications for stretching intensity) due to the subjective influence of the participants. Future studies may therefore use isokinetic dynamometer or other devices which enable a quantification of passive resistance of the muscle to objectively quantify stretching intensity. Finally, while we tested and stretched muscle and fascia stiffness in the gastrocnemius, on the one hand, the soleus stiffness values could also have been affected. On the other hand, depending on the reached value in the KtW, due to flexing the knee joint, the flexibility test focus might shift from the gastrocnemius to the soleus muscle, which could explain the lack of significant effects in this study.

## Conclusion

Stretching acutely reduces fascia stiffness and the magnitude of this decrease is comparable to that in the skeletal muscle. When compared to dynamic application, static stretch seems to have superior effects. A small association exists between changes of fascia stiffness and ROM increases, meaning that fascial but not muscle stiffness reductions could be a contributor of ROM improvements.

## Outlook

Most previous works neglected the role of connective tissue properties when investigating flexibility increases in response to stretching. Our study was the first that explored the acute effects of different stretching types on muscle and fascia stiffness and related these to ROM changes. Since we were able to show fascial tissue to respond to mechanical overload acutely, future studies, both on acute and chronic effects, should consider a more holistic approach instead of focusing exclusively on muscle parameters.

## Data Availability

Original data can be provided due to reasonable request.
